# KIAA1429 protects hepatocellular carcinoma cells from ferroptotic cell death with a m^6^A‐dependent posttranscriptional modification of SLC7A11

**DOI:** 10.1111/jcmm.17997

**Published:** 2023-10-13

**Authors:** Houhong Wang, Wenli Chen, Yayun Cui, Huihui Gong, Heng Li

**Affiliations:** ^1^ Department of General Surgery The Affiliated Bozhou Hospital of Anhui Medical University Bozhou Anhui China; ^2^ Division of Life Sciences and Medicine, Department of Cancer Radiotherapy, The First Affiliated Hospital of USTC University of Science and Technology of China (Anhui Provincial Cancer Hospital) Hefei Anhui China; ^3^ Faculty of Health and Life Sciences Oxford Brookes University Oxford United Kingdom; ^4^ Department of Comprehensive Surgery Anhui Provincial Cancer Hospital, West District of The First Affiliated Hospital of USTC Hefei Anhui China

**Keywords:** ferroptosis, hepatocellular carcinoma, KIAA1429, lipid peroxidation, m^6^A modification, SLC7A11

## Abstract

N^6^‐methyladenosine (m^6^A) modification represents the most abundant internal methylation of eukaryotic RNAs. KIAA1429 acts as a key component of the m^6^A methyltransferase complex, but its function and mechanism in ferroptotic cell death of hepatocellular carcinoma (HCC) are barely defined. We found that KIAA1429 suppression triggered ferroptosis in HCC cells according to increased cell death, iron and MDA levels, C11‐BODIPY‐positive cells, ROS production and decreased GSH level. Ferroptosis inhibitors ferrostatin‐1 (0.5 μM) and liproxstatin‐1 (10 μM) blocked KIAA1429 suppression‐induced ferroptosis of HCC cells. In addition, overexpressed KIAA1429 notably heightened the activity of cystine/glutamate antiporter (SLC7A11). SLC7A11 up‐regulation partially hindered KIAA1429 inhibition‐mediated ferroptosis of HCC cells. The regulation SLC7A11 by KIAA1429 was attenuated by global m^6^A inhibitor cycloleucine (40 μM). RNA immunoprecipitation confirmed the binding of KIAA1429 to m^6^A on SLC7A11 transcript. Additionally, it was proven that KIAA1429 inhibition mitigated HCC growth in subcutaneous xenograft mice through SLC7A11. Altogether, our findings first propose that KIAA1429 protects HCC cells from ferroptosis with a m^6^A‐dependent post‐transcriptional modification of SLC7A11 and offer a novel insight into the dysregulated epi‐transcriptomics in the context of HCC.

## 1 INTRODUCTION

Liver cancer is a main cause of death globally, being the seventh most diagnosed cancer and the third major cause of cancer‐related death, with about 905,677 newly diagnosed cases and 830,180 deaths in 2020 according to the latest global statistics.^1^ Hepatocellular carcinoma (HCC) comprises 75%–85% of primary liver cancer cases, with five‐year survival rate of approximately 15%.^2^ Hepatocellular carcinoma is an extremely heterogeneous malignancy with limited treatment options due to multiple factors that contribute to its pathophysiology, hepatitis B/C virus infection, underlying liver diseases, etc^3^ Curative surgical resection remains the standard of care for eligible patients.^4^ Nonetheless, about 50% of patients receive systemic treatment during their disease course, particularly in the advanced stages.^5^ Hence, to clarify the molecular mechanisms of HCC is crucial for the development of effective treatment options.

N^6^‐methyladenosine (m^6^A) RNA modification acts as the most abundant post‐transcriptional machinery in eukaryotic mRNAs, which is a reversible process controlled by methyltransferases, demethylases and m^6^A‐binding proteins.^6^, ^7^ M^6^A modification plays a unique role in important physiological liver functions and diverse liver diseases, particularly HCC.^8^ KIAA1429 is a key component of the m^6^A methyltransferase complex. Evidence has demonstrated that KIAA1429 triggers HCC progression with a m^6^A‐dependent post‐transcriptional modification of GATA3.^9^ In addition, KIAA1429‐mediated circDLC1 mitigates MMP1‐triggered HCC progression by interacting with HuR.^10^ Ferroptosis is an iron‐dependent type of cell death with extensive lipid peroxidation on the cellular membrane,^11^ which is morphologically and mechanistically distinct from other forms of regulated cell death.^12^ As a unique cell death form, ferroptosis has attracted great interest in the cancer research community.^13^ Recently, remarkable progress has been achieved in comprehending the function of ferroptosis in tumour biology and HCC treatment. Prevention of ferroptosis drives resistance to sorafenib in HCC.^14^ Hao et al.^15^ designed engineered exosomes for targeted and potently inducing ferroptosis in HCC through chemo‐photodynamic treatment. Altogether, targeting ferroptosis may offer a novel therapeutic opportunity for the treatment of HCC refractory to conventional therapy. Nevertheless, the function and mechanism of KIAA1429 in regulating ferroptotic cell death of HCC are barely defined. SLC7A11, a cystine/glutamate antiporter, acts as importing cystine for glutathione biosynthesis and antioxidant defence, which exhibits up‐regulation in HCC.^16^ As a ferroptosis suppressor, SLC7A11 up‐regulation facilitates tumour growth partially via attenuating ferroptosis.^17^ However, the regulatory mechanisms of SLC7A11 in the context of HCC remain unclear. Evidence has proved that m^6^A modification affects ferroptosis in diseased liver through post‐transcriptionally regulated gene expression. For instance, hypoxic microenvironment blocks ferroptotic cell death of HCC cells through mitigating METTL14‐ and YTHDF2‐dependent inhibition of SLC7A11.^18^ M^6^A modification modulates ferroptotic cell death via autophagy signalling in hepatic stellate cells.^19^ M^6^A RNA methylation is essential for dihydroartemisinin to mitigate liver fibrosis by heightening ferroptotic cell death of hepatic stellate cells.^20^ Herein, we propose a novel insight that KIAA1429 protects HCC cells from ferroptosis with m^6^A‐dependent post‐transcriptional modification of SLC7A11.

## 2 MATERIALS AND METHODS

### 2.1 Cell culture, lentiviral vectors and infection

Huh‐7 and SK‐Hep1 cell lines were cultivated in Dulbecco's Modified Eagle medium (HyClone) supplemented with 10% foetal bovine serum (HyClone) at 37°C and 5% CO_2_. KIAA1429‐targeting short hairpin RNA (sh‐KIAA1429) or nonspecific control (sh‐NC) was constructed utilising pGLVU6/GFP lentiviral vectors. LV11‐CMV‐MCS‐hPGK‐GFP‐Puro vectors were applied for constructing KIAA1429‐ and SLC7A11‐overexpressing lentiviruses (Lv‐KIAA1429 and ‐SLC7A11). Above lentiviruses were purchased from GenePharma. Hepatocellular carcinoma ferroptosis was mitigated through administration with 0.5 μM ferrostatin‐1 (Fer‐1; MedChemExpress) or 10 μM liproxstatin‐1 (Lip‐1; MedChemExpress) for 24 h. In addition, cells were exposed to 40 μM m^6^A methylation inhibitor cycloleucine for 24 h. Equivalent dose of DMSO was utilised as controls.

### 2.2 Quantitative RT‐PCR

Total RNA isolation was conducted with RNeasy Mini kit (QIAGEN), and RNA content was evaluated through NanoDrop 2000 spectrophotometer (Thermo Fisher). Qualified RNA was utilised for synthesising cDNA through EasyScript First‐Strand cDNA Synthesis SuperMix (TransGen). Primer sequences included: KIAA1429, 5′‐AAGTGCCCCTGTTTTCGATAG‐3′ (forward) and 5′‐ACCAGACCATCAGTATTCACCT‐3′ (reverse); GAPDH, 5′‐CTGGGCTACACTGAGCACC‐3′ (forward) and 5′‐AAGTGGTCGTTGAGGGCAATG‐3′ (reverse). Quantitative RT‐PCR was assayed with SuperScript IV One‐Step RT‐PCR System (Thermo Fisher) together with SYBR Green PCR Mastermix (Solarbio). Relative mRNA level was computed with $2^{-∆∆\mathrm{CT}}$ approach, with GAPDH as a housekeeping gene.

### 2.3 Immunoblotting

Cell pellets were lysed with radioimmunoprecipitation lysis (Solarbio) on ice for 30 min, followed by centrifugation at 12,000 *g* at 4°C for 10 min. Protein concentration was assayed with Bradford assay (Solarbio), and extracted protein was resolved through SDS‐PAGE and transferred to polyvinylidene difluoride membranes (Millipore, USA). The membranes were sealed by 5% BSA in TBST, followed by incubation with primary antibodies of KIAA1429 (1/1000; ab271136; Abcam), GAPDH (1/2000; ab37168), SLC7A11 (1/2000; ab175186), FSP1 (1/1000; ab197896), DHODH (1/1000; ab174288), GPX4 (1/1000; ab252833) or ACSL4 (1/10000; ab155282) along with secondary antibodies (1/10000; 31460; Thermo Fisher). The immunoreactivity was captured through chemiluminescence imaging system (Solarbio). Afterwards, optical density was quantified with ImageJ software (Bio‐Rad).

### 2.4 Flow cytometric analysis

Apoptotic cells were detected by Annexin V‐PE apoptosis detection kit (Thermo Fisher) following the manufacturer's specifications. Following trypsinization, cells were harvested and washed with PBS. They were dyed with Annexin V‐PE staining reagent for 15 min at room temperature. Apoptotic cells were measured utilising BD FACSCalibur™ (BD).

### 2.5 TdT mediated dUTP Nick End Labeling (TUNEL) assay

Apoptosis was detected with TUNEL Apoptosis Detection Kit (Alexa Fluor 640; YEASEN). Cells were inoculated into an 18‐mm cover glass coverslip in a 12‐well plate and fixed by pre‐cold acetic acid/ethanol reagent. Afterwards, they were washed with PBS and dyed with TUNEL reagent. Sections were mounted, and fluorescence images were captured.

### 2.6 Iron assay

Cellular iron level was measured with iron assay kit (BioAssay Systems). Cells were homogenised through five volumes of iron assay buffer followed by 13,000 *g* centrifugation for 10 min at 4°C. Afterwards, incubation of iron reducer together with supernatant mixtures was conducted for 30 min. Samples were then cultured with iron probe for 60 min away from light. The absorbance values were measured at 593 nm.

### 2.7 Lipid peroxidation assay

Malonaldehyde (MDA) level was assayed with lipid peroxidation assay kit (BioVision). Cell lysate supernatant was harvested and incubated with 600 μL thiobarbituric acid reagent at 95°C for 60 min. Each reaction mixture was transferred 200 μL to a 96‐well plate, followed by MDA measurement.

Cells were dyed with C11‐BODIPY (Thermo Fisher) for 30 min following the manufacturer's specifications. Under the well without C11‐BODIPY as control, cells were washed and detected through flow cytometer (BD).

### 2.8 Reactive oxygen species (ROS) assay

Intracellular ROS level was assayed with by 2′,7′‐dichlorofluorescin diacetate (Sigma‐Aldrich). Briefly, cells were dyed with 5 μM 2′,7′‐dichlorofluorescin diacetate in PBS for 5 min, which were subjected to flow cytometer (BD) within 15 min.

### 2.9 Glutathione (GSH) assay

Cells were washed with cold PBS, and GSH level was measured with GSH assay kit (BioAssay Systems). Three volumes of 5% sodium sulphate aqueous (SSA) solution were added, followed by 10,000 *g* centrifugation for 10 min. The supernatant was harvested to determine the GSH level. Samples were incubated with 150 μL GSH working mixture at room temperature for 5 min. Afterwards, 50 μL NADPH solution was added. The GSH level was measured at 412 nm.

### 2.10 Immunofluorescent staining

A 2 × 10^5^ cells were inoculated onto cell‐climbing slices in a 24‐well plate. After fixing with 4% paraformaldehyde, cells were permeabilized with 0.5% Triton X‐100 and sealed with 10% goat serum. Primary antibody of SLC7A11 (1/500; ab37185) was incubated overnight at 4°C. Next day, cells were incubated with secondary antibody for 1.5 h at room temperature. Cell‐climbing slices were mounted and acquired under a confocal laser scanning microscopy (Leica).

### 2.11 RNA immunoprecipitation (RIP) assay

RNA immunoprecipitation was assayed with EZ‐Magna RIP kit (Millipore). Cells were resuspended with 200 μL RIP lysis reagent, and magnetic bead was coated with antibodies (Ab‐Flag or Ab‐m^6^A; Abcam) for immunoprecipitation. IgG served as control. Following centrifugation, 100 μL supernatants were discarded. Bead‐antibody complexes within RIP immunoprecipitation reagent were incubated with each tube at 4°C overnight. Immunoprecipitation was resuspended with 150 μL proteinase K buffer (Millipore), followed by incubation at 55°C lasting 30 min. Following centrifugation, the tube was added onto magnetic separator for collecting the supernatants, followed by RNA extraction and precipitation.

### 2.12 Human specimens

Hepatocellular carcinoma and paired adjacent normal tissues (*n* = 3) were acquired from patients who received surgical resection in the Affiliated Hangzhou First People's Hospital, Zhejiang University School of Medicine. Fresh tissue specimens were stored in liquid nitrogen. The protocols of our study were carried out following the Ethical Review Committees of the Affiliated Bozhou Hospital of Anhui Medical University (2022‐17). In line with the policies of the committee, the written informed consent was obtained from all patients.

### 2.13 Animal studies

Male BALB/c nude mice (6‐week‐old; BEIJING HFK BIOSCIENCE, China) were raised following an aseptic‐specified pathogen‐free condition. All surgical procedures were conducted under sodium pentobarbital anaesthesia. 5 × 10^5^ Huh‐7 cells with control, sh‐KIAA1429, or sh‐KIAA1429 + Lv‐SLC7A11 were injected subcutaneously into the right axilla of each mouse. Tumour volume was measured weekly, and computed as length × width^2^ × 0.5. All animal experiments were authorised by the Institutional Animal Care and Use Committee of the Affiliated Bozhou Hospital of Anhui Medical University (2022‐17).

### 2.14 Immunohistochemistry

Tumour tissues were fixed in formalin and embedded in paraffin. 4‐μm‐thick sections were mounted, dewaxed in xylene and rehydrated with a gradient of ethanol and distilled water. The sections were incubated with primary antibody against KIAA1429 or SLC7A11 overnight at 4°C and then probed with HRP anti‐rabbit IgG antibody (1/200; ab288151; Abcam). KIAA1429 and SLC7A11 expressions were quantified with ImageJ software.

### 2.15 Bioinformatics analysis

Based on the GEPIA online tool, the mRNA expression of KIAA1429 and SLC7A11 was measured in TCGA HCC (*n* = 369) and normal tissues (*n* = 160). In addition, TCGA HCC patients were divided into two groups according to the median value of KIAA1429 or SLC7A11, and overall survival analysis between groups was then conducted. The correlation between KIAA1429 and SLC7A11 among TCGA HCC patients was conducted via Pearson's test.

### 2.16 Statistics

Each experiment was independently repeated at least three times. Data were expressed as the mean ± standard error. All statistical analyses were conducted utilising GraphPad Prism 8.0.1 software. Statistical comparisons between multiple groups were implemented with one‐ or two‐way anova. Statistical significance was indicated by *p* values < 0.05.

## 3 RESULTS

### 3.1 Ferroptosis inhibitor Fer‐1 or Lip‐1 blocks KIAA1429 suppression‐induced death of HCC cells

**FIGURE 1:**
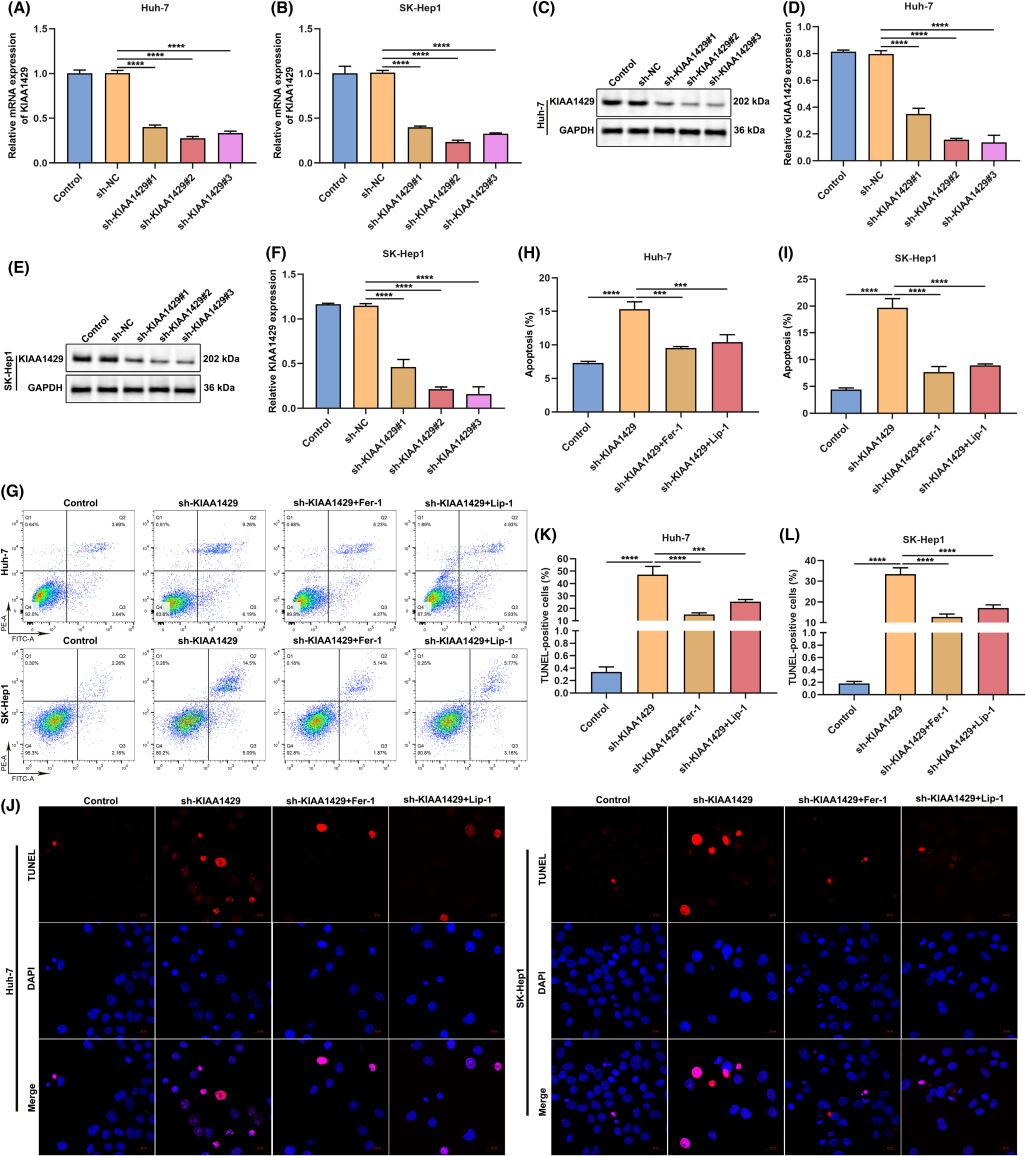
Ferroptosis inhibitor Fer‐1 or Lip‐1 hinders KIAA1429 inhibition‐induced death of HCC cells. (A, B) The interference effect of sh‐KIAA1429 in Huh‐7, and SK‐Hep1 cells through quantitative RT‐PCR. (C–F) Protein level of KIAA1429 in sh‐KIAA1429‐transfected HCC cells, with GAPDH as a loading control. (G–I) Cellular death level was tested through flow cytometric analysis in Huh‐7 together with SK‐Hep1 cells of sh‐KIAA1429 transfection, with or without 0.5 μM Fer‐1 or 10 μM Lip‐1 administration. (J–L) Cellular death was measured via adopting TUNEL staining in HCC cells of sh‐KIAA1429 transfection, with or without 0.5 μM Fer‐1 or 10 μM Lip‐1 administration. Scale bar, 20 μm. ***p < 0.001; ****p < 0.0001.

To silence KIAA1429 expression, the current study established lentivirus‐carried shRNAs against KIAA1429 (sh‐KIAA1429) through transfection to two HCC cells (Huh‐7 and SK‐Hep1). Afterwards, we measured the knockdown effects, and the results confirmed that KIAA1429 level was observably mitigated by sh‐KIAA1429 in Huh‐7 and SK‐Hep1 cells (Figure 1A–F). Apoptotic level of HCC cells was tested via adopting flow cytometric analysis and TUNEL staining. KIAA1429 suppression by sh‐KIAA1429 dramatically heightened death of Huh‐7 and SK‐Hep1 cells (Figure 1G–L). Ferroptosis is an iron‐dependent type of regulated cell death, with the accumulated lipid peroxides to lethal amounts.^21^ To prevent HCC ferroptosis, HCC cells were administrated 0.5 μM Fer‐1 or 10 μM Lip‐1 lasting 24 h. Consequently, both ferroptosis inhibitors Fer‐1 and Lip‐1 notably weakened the effect of sh‐KIAA1429 on death of Huh‐7 and SK‐Hep1 cells. Altogether, ferroptosis inhibitors Fer‐1 and Lip‐1 may block KIAA1429 suppression‐induced HCC cell death.

### 3.2 KIAA1429 suppression induces HCC cell death via ferroptosis

**FIGURE 2:**
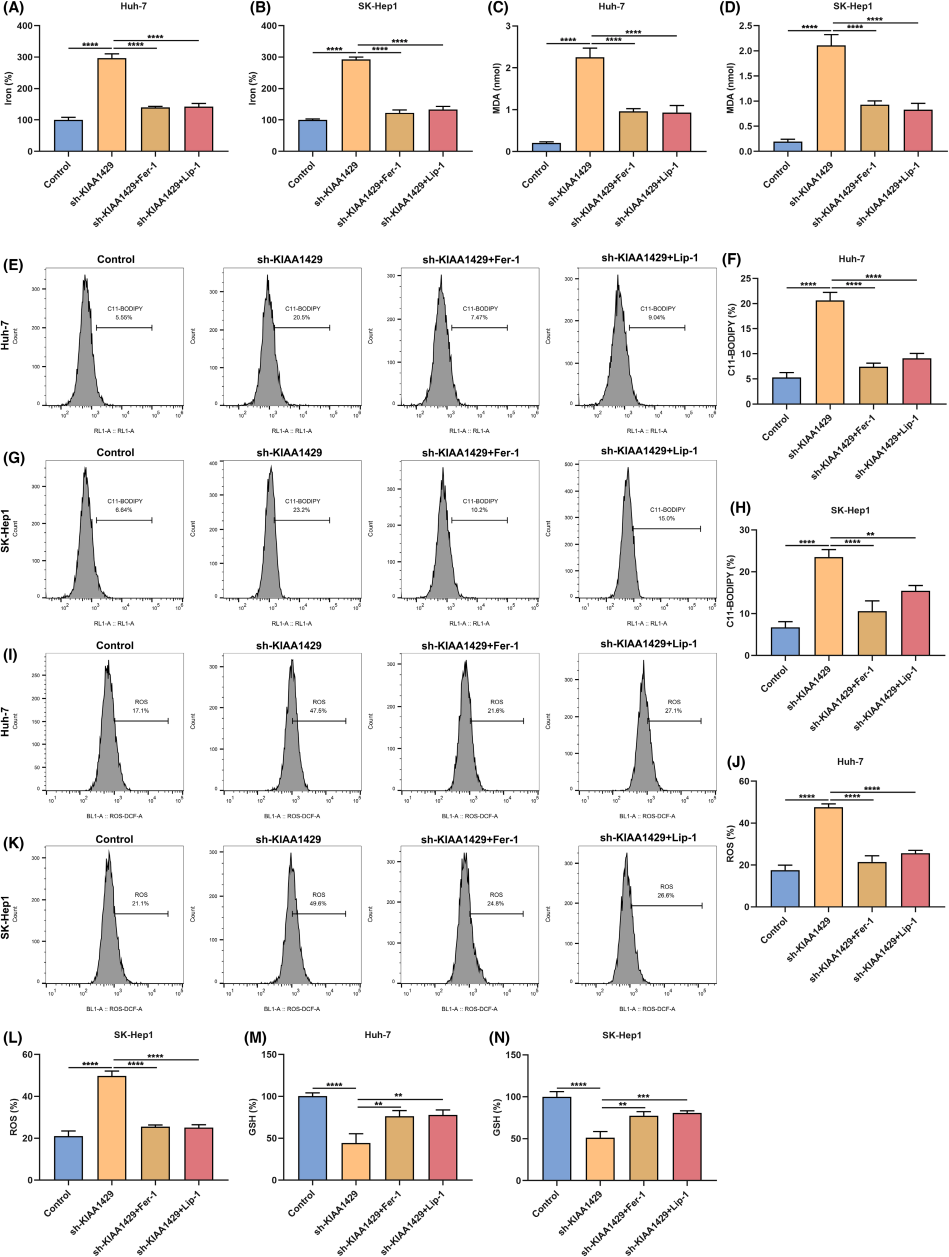
KIAA1429 suppression induces HCC cell death via ferroptosis. (A, B) Iron level in Huh‐7 together with SK‐Hep1 cells of sh‐KIAA1429 transfection, with or without 0.5 μM Fer‐1 or 10 μM Lip‐1 administration. (C, D) The amount of MDA in HCC cells of sh‐KIAA1429 transfection, with or without 0.5 μM Fer‐1 or 10 μM Lip‐1 treatment. (E–H) C11‐BODIPY‐positive HCC cells under sh‐KIAA1429 transfection, with or without 0.5 μM Fer‐1 or 10 μM Lip‐1 treatment through flow cytometric analysis. (I–L) Intracellular ROS level in HCC cells of sh‐KIAA1429 transfection, with or without 0.5 μM Fer‐1 or 10 μM Lip‐1 treatment via adopting flow cytometric analysis. (M, N) GSH level in HCC cells under sh‐KIAA1429 transfection, with or without 0.5 μM Fer‐1 or 10 μM Lip‐1 administration. **p < 0.01; ***p < 0.001; ****p < 0.0001.

The present study puts forward the hypothesis that KIAA1429 inhibition may induce cellular death through ferroptosis. Iron level of Huh‐7 and SK‐Hep1 cells was attenuated by sh‐KIAA1429 (Figure 2A,B). Nonetheless, Fer‐1 and Lip‐1 blocked the inhibitory effect of KIAA1429 deficiency on iron level of HCC cells. MDA (the end product of lipid peroxidation) presented an elevated amount in KIAA1429‐inhibited cells, indicating an enhanced lipid oxidation level and oxidative damage (Figure 2C,D). Rather, Fer‐1 and Lip‐1 partially reversed the amount of MDA triggered by KIAA1429 suppression. Oxidized C11‐BODIPY is also usually utilised as a ferroptotic marker, which was measured through immunolabelled oxidized C11‐BODIPY of flow cytometric analysis. As a result, C11‐BODIPY‐positive percentage was memorably elevated by sh‐KIAA1429, which was blocked by Fer‐1 and Lip‐1 (Figure 2E–H). Except for lipid peroxidation, ROS generation is a key mechanism of ferroptosis. Next, intracellular ROS level was tested via flow cytometric analysis. Consequently, KIAA1429‐inhibited cells displayed higher ROS production (Figure 2I–L). Oppositely, Fer‐1 and Lip‐1 lowered sh‐KIAA1429‐mediated ROS accumulation. In addition, intracellular GSH level was attenuated by KIAA1429 inhibition, which was elevated by Fer‐1 and Lip‐1 (Figure 2M,N). Above data demonstrated that KIAA1429 inhibition enabled to trigger HCC cell death through ferroptosis.

### 3.3 KIAA1429 targets SLC7A11 during ferroptotic cell death in HCC cells

**FIGURE 3:**
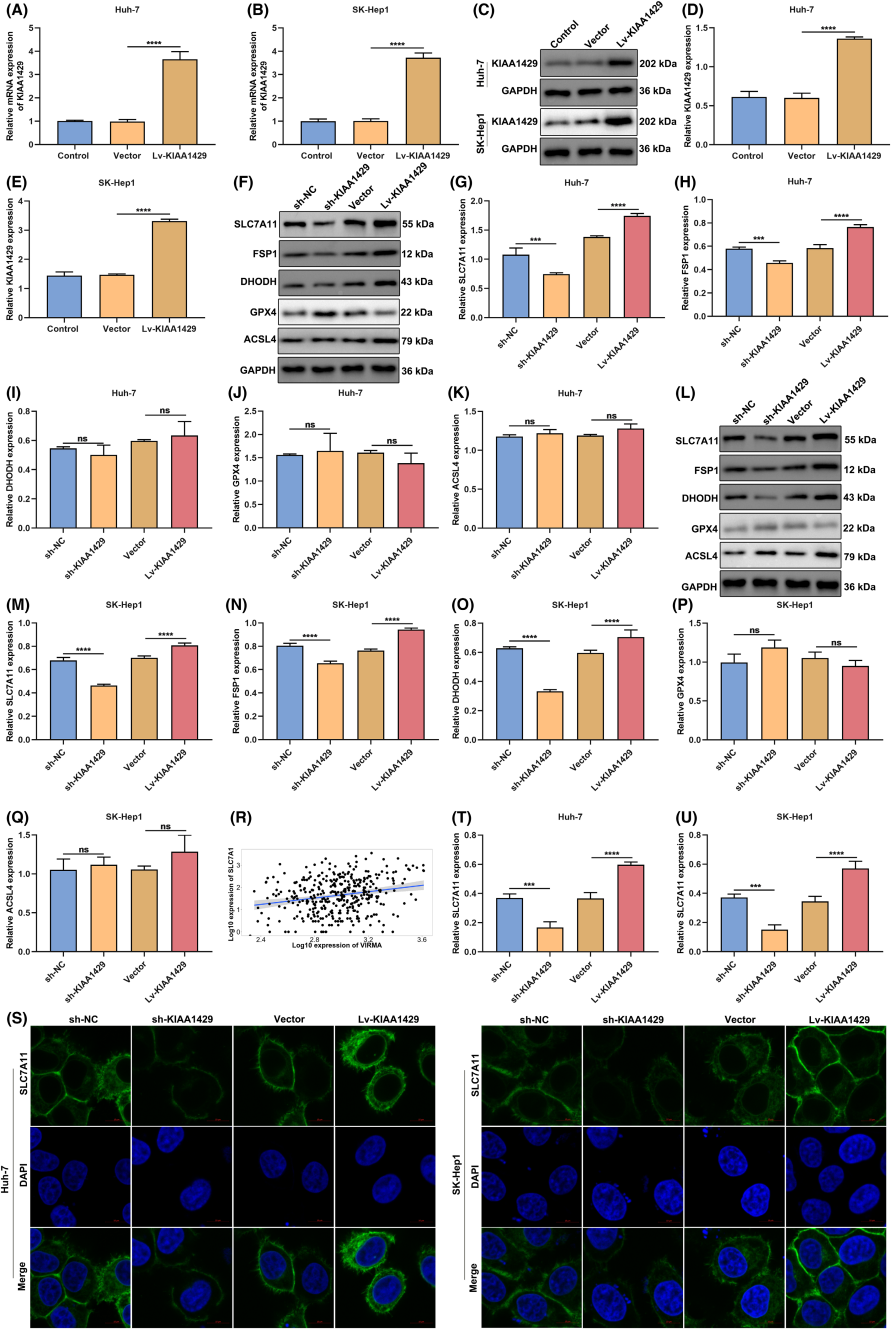
KIAA1429 targets SLC7A11 during ferroptotic cell death in HCC cells. (A, B) Overexpression effect of Lv‐KIAA1429 in Huh‐7 and SK‐Hep1 cells via quantitative RT‐PCR. (C–E) Protein level of KIAA1429 in Lv‐KIAA1429‐transfected HCC cells, with GAPDH as a loading control. (F–K) Protein levels of SLC7A11, FSP1, DHODH, GPX4 and ACSL4 in Huh‐7 cells with sh‐KIAA1429 or Lv‐KIAA1429. (L–Q) Protein levels of SLC7A11, FSP1, DHODH, GPX4 and ACSL4 in SK‐Hep1 cells with sh‐KIAA1429 or Lv‐KIAA1429. (R) Correlation analysis of KIAA1429 with SLC7A11 across HCC patients. (S–U) Immunofluorescent staining of SLC7A11 level in Huh‐7 together with SK‐Hep1 cells of sh‐KIAA1429 or Lv‐KIAA1429. Scale bar, 10 μm. ***p < 0.001; ****p < 0.0001; ns: no significance.

Lentiviral vectors carrying KIAA1429 gene (Lv‐KIAA1429) were transfected into Huh‐7 or SK‐Hep1 cells. It was verified that KIAA1429 was observably overexpressed (Figure 3A–E). Afterwards, ferroptosis‐related genes SLC7A11, FSP1, DHODH, GPX4 and ACSL4 were examined in Huh‐7 and SK‐Hep1 cells with KIAA1429 knockdown or overexpression. Both in Huh‐7 and SK‐Hep1 cells, SLC7A11 and FSP1 levels were markedly lowered by sh‐KIAA1429 (Figure 3F–Q). Oppositely, overexpressed SLC7A11 and FSP1 were observed in Lv‐KIAA1429‐mediated cells. In addition, a remarkably positive interaction between KIAA1429 and SLC7A11 was confirmed in TCGA‐LIHC cohort (Figure 3R). Immunofluorescent staining also demonstrated the decreased level of SLC7A11 by sh‐KIAA1429 in Huh‐7/SK‐Hep1 cells, with an opposite effect by Lv‐KIAA1429 (Figure 3S–U). Altogether, KIAA1429 acted as a ferroptotic suppressor by modulating SLC7A11 expression.

### 3.4 KIAA1429 suppression induces HCC cell death partially through modulating SLC7A11 expression

**FIGURE 4:**
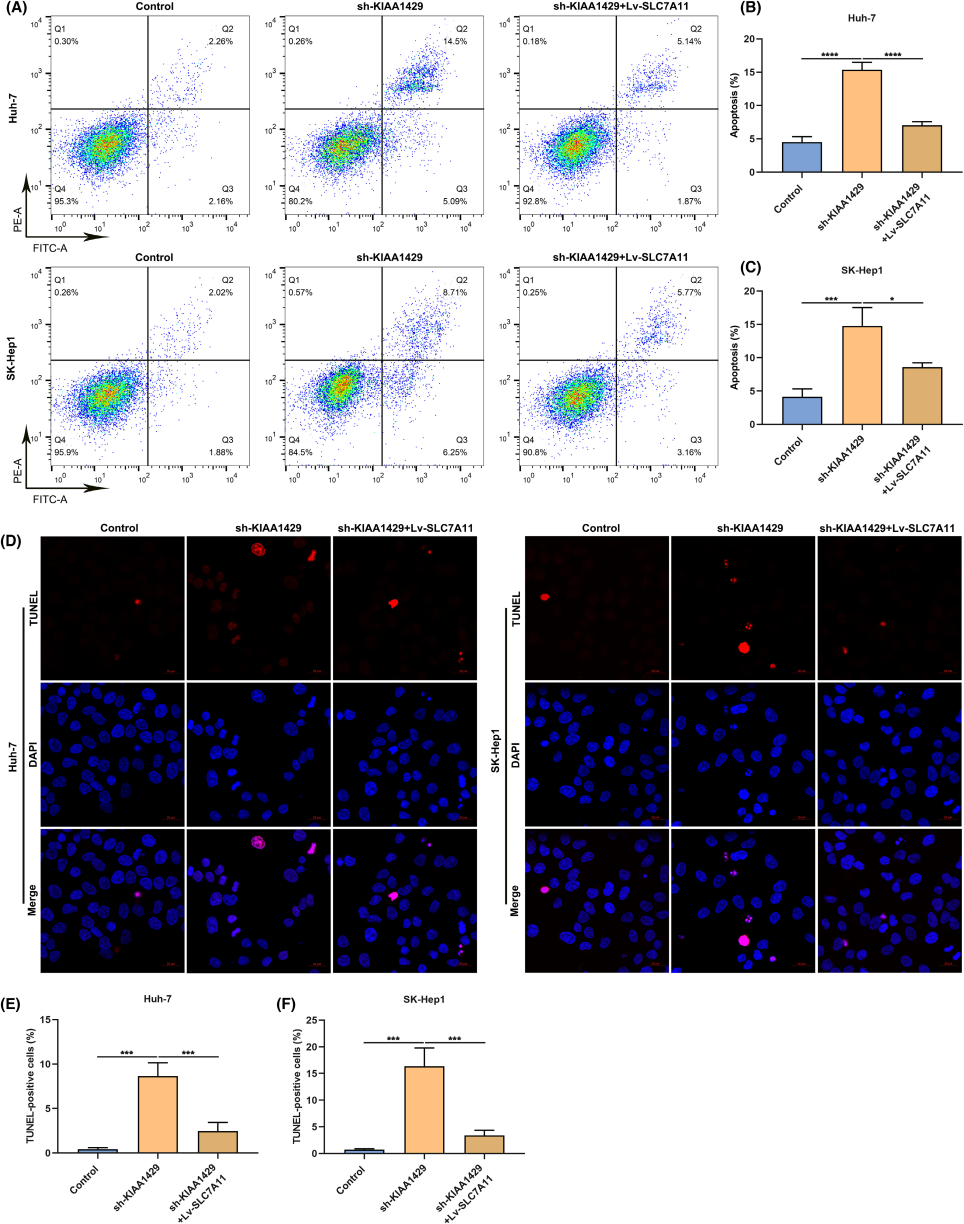
KIAA1429 suppression induces HCC cell death through modulating SLC7A11 expression. (A–C) Cellular death of Huh‐7 together with SK‐Hep1 cells of sh‐KIAA1429 together with/without Lv‐SLC7A11 through flow cytometric analysis. (D‐F) TUNEL staining of cellular death of HCC cells with sh‐KIAA1429 together with/without Lv‐SLC7A11. Scale bar, 20 μm. *p < 0.05; ***p < 0.001; ****p < 0.0001.

Cystine/glutamate antiporter SLC7A11 is responsible for importing cystine for glutathione biosynthesis and antioxidant defence,^22^ and its up‐regulation contributes to HCC growth partially via mitigating ferroptotic cell death.^23^ Cell death was notably induced by sh‐KIAA1429 in Huh‐7/SK‐Hep1 cells, which was restraint by lentiviral vectors carrying SLC7A11 gene (Lv‐SLC7A11) in accordance with the results from flow cytometric analysis (Figure 4A–C) and TUNEL staining (Figure 4D–F).

### 3.5 KIAA1429 suppression triggers ferroptosis of HCC cells partially through SLC7A11

**FIGURE 5:**
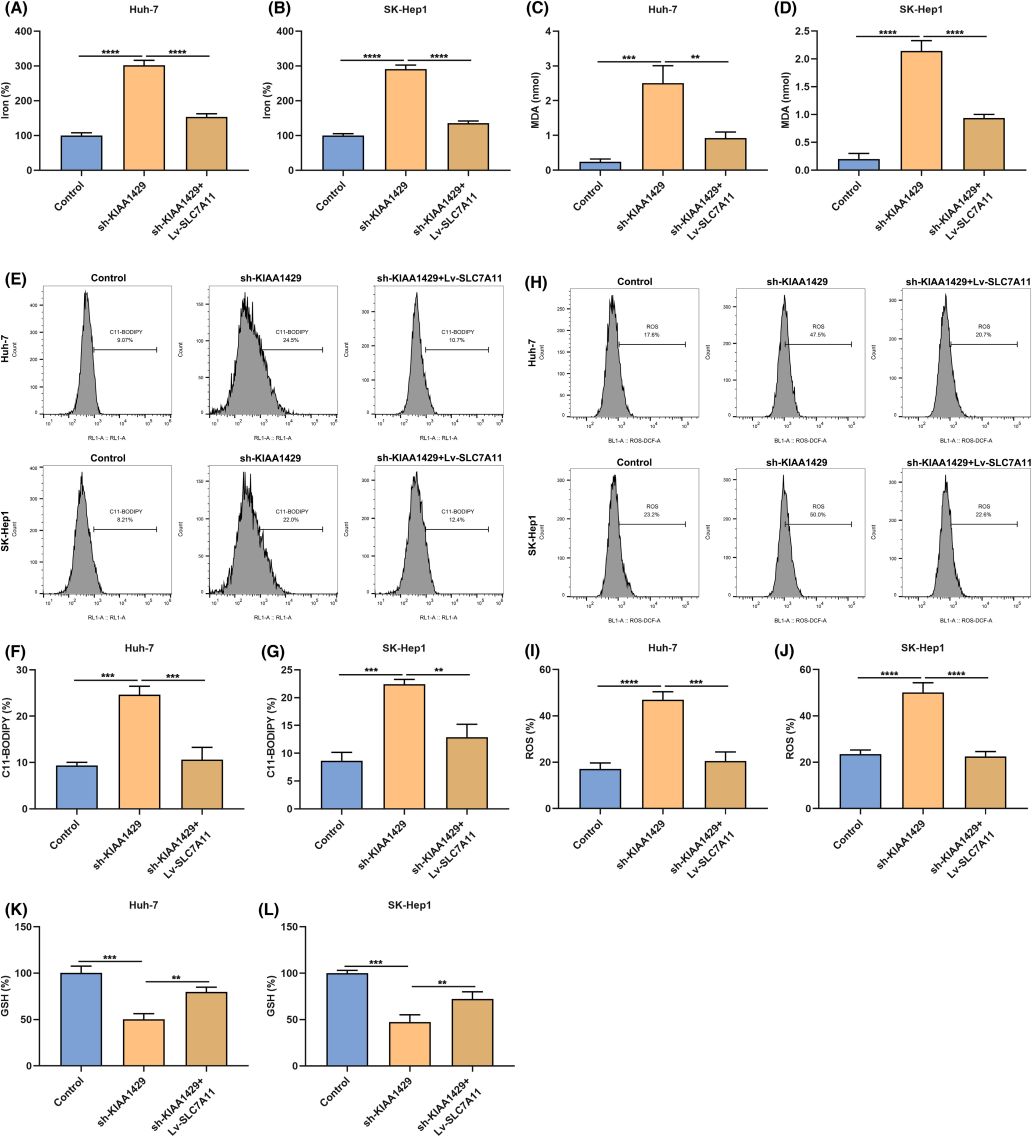
KIAA1429 suppression triggers ferroptosis of HCC cells partially through SLC7A11. (A, B) Iron level in Huh‐7 together with SK‐Hep1 cells of sh‐KIAA1429 together with/without Lv‐SLC7A11. (C, D) The amount of MDA in HCC cells of sh‐KIAA1429 together with/without Lv‐SLC7A11. (E–H) C11‐BODIPY‐positive HCC cells under sh‐KIAA1429 together with/without Lv‐SLC7A11 via adopting flow cytometric analysis. (I–J) Intracellular ROS level in HCC cells with sh‐KIAA1429 together with/without Lv‐SLC7A11 utilising flow cytometric analysis. (K, L) GSH level in HCC cells under sh‐KIAA1429 together with/without Lv‐SLC7A11. **p < 0.01; ***p < 0.001; ****p < 0.0001.

Both in Huh‐7 and SK‐Hep1 cells, sh‐KIAA1429 notably increased iron levels and the amount of MDA, which were whittled by Lv‐SLC7A11 (Figure 5A–D). In addition, C11‐BODIPY‐positive Huh‐7/SK‐Hep1 cells were induced by sh‐KIAA1429 (Figure 5E–H). In contrast, Lv‐SLC7A11 observably lowered C11‐BODIPY‐positive cells. As illustrated in Figure 5I,J, sh‐KIAA1429‐mediated ROS accumulation in Huh‐7/SK‐Hep1 cells was decreased by Lv‐SLC7A11. KIAA1429‐inhibited HCC cells displayed lower GSH level, which was elevated by Lv‐SLC7A11 (Figure 5K,L). In conclusion, KIAA1429 inhibition triggered ferroptosis of HCC cells partially through modulating SLC7A11 expression.

### 3.6 KIAA1429 up‐regulates SLC7A11 expression in HCC cells in an m^6^A‐dependent manner

**FIGURE 6:**
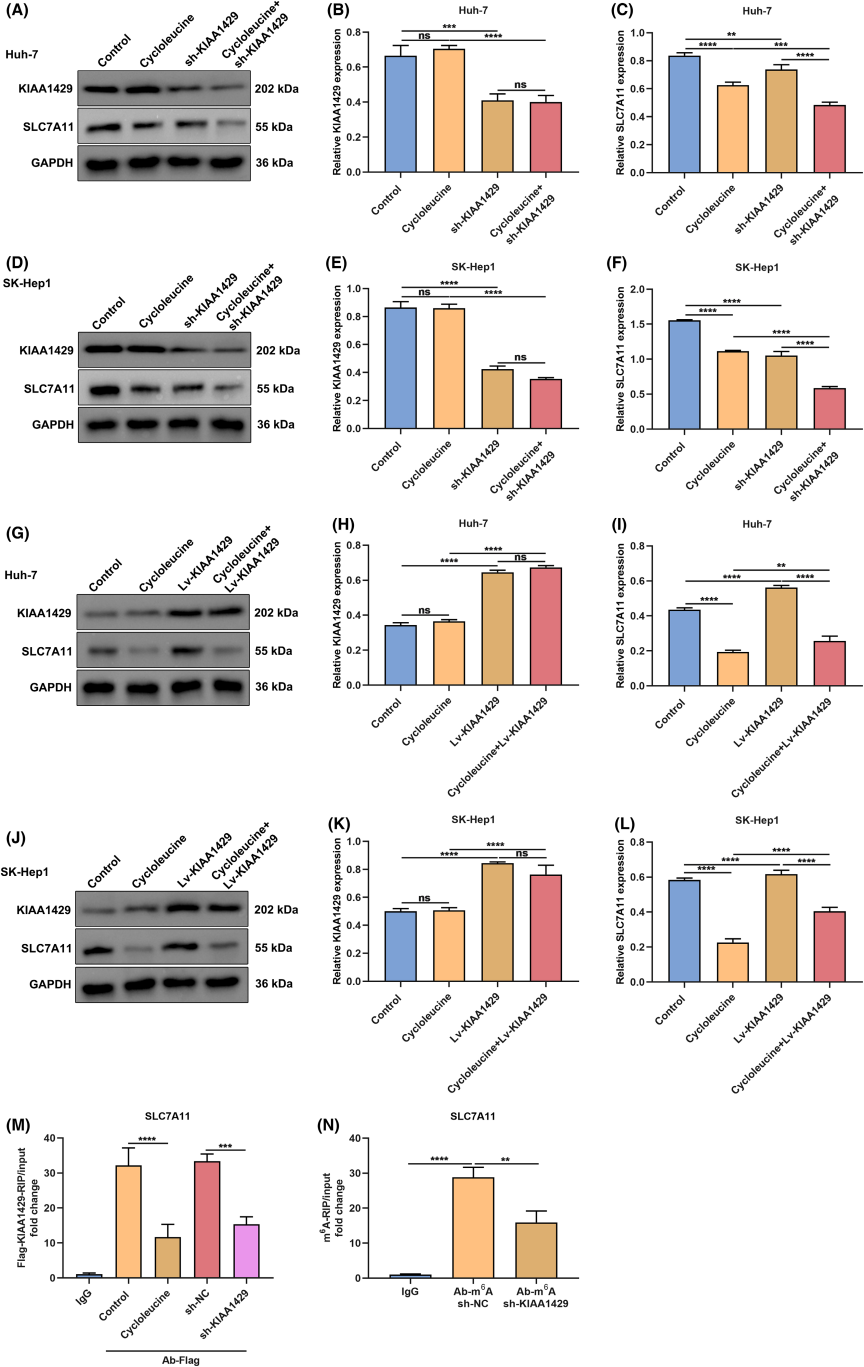
KIAA1429 up‐regulates SLC7A11 expression in HCC cells in an m^6^A‐dependent manner. (A–C) Immunoblotting of KIAA1429 and SLC7A11 levels in Huh‐7 cells with sh‐KIAA1429 together with/without 40 μM cycloleucine. (D–F) Immunoblotting of KIAA1429 and SLC7A11 levels in SK‐Hep1 cells with sh‐KIAA1429 together with/without 40 μM cycloleucine. (G–I) Immunoblotting of KIAA1429 and SLC7A11 levels in Huh‐7 cells with Lv‐SLC7A11 together with/without 40 μM cycloleucine. (J–L) Immunoblotting of KIAA1429 and SLC7A11 levels in SK‐Hep1 cells with Lv‐SLC7A11 together with/without 40 μM cycloleucine. (M) Flag‐KIAA1429‐RIP of enrichment level of SLC7A11 mRNA under sh‐KIAA1429 together with/without 40 μM cycloleucine. (N) M^6^A‐RIP of enrichment level of SLC7A11 mRNA under sh‐NC or sh‐KIAA1429. **p < 0.01; ***p < 0.001; ****p < 0.0001; ns: no significance.

Then, the current study observed the mechanisms under KIAA1429 modulates SLC7A11 in HCC cells. 40 μM global m^6^A inhibitor cycloleucine was utilised for investigating whether KIAA1429 regulated downstream expression through m^6^A RNA modification. As a result, SLC7A11 expression declined to a low level in Huh‐7/SK‐Hep1 cells with KIAA1429 suppression or cycloleucine administration (Figure 6A–F). Lv‐KIAA1429 observably up‐regulated SLC7A11 expression, which was affected by cycloleucine (Figure 6G–L). For verifying the direct binding between KIAA1429 and SLC7A11 mRNA transcripts, this study utilised Flag‐KIAA1429 plasmids and Ab‐Flag for precipitating mRNA, followed by quantitative RT‐PCR. The enrichment level of SLC7A11 mRNA was tested through RIP/input. Consequently, lower enrichment levels were observed in cycloleucine and sh‐KIAA1429 groups in contrast to control group (Figure 6M), demonstrating that m^6^A RNA modification might be essential for binding. The m^6^A‐RIP results showed that sh‐KIAA1429 group displayed lower enrichment level of SLC7A11 mRNA in comparison to control group (Figure 6N). Hence, KIAA1429 may up‐regulate SLC7A11 expression in HCC cells in an m^6^A‐dependent manner.

### 3.7 KIAA1429 inhibition mitigates HCC growth in vivo through SLC7A11

**FIGURE 7:**
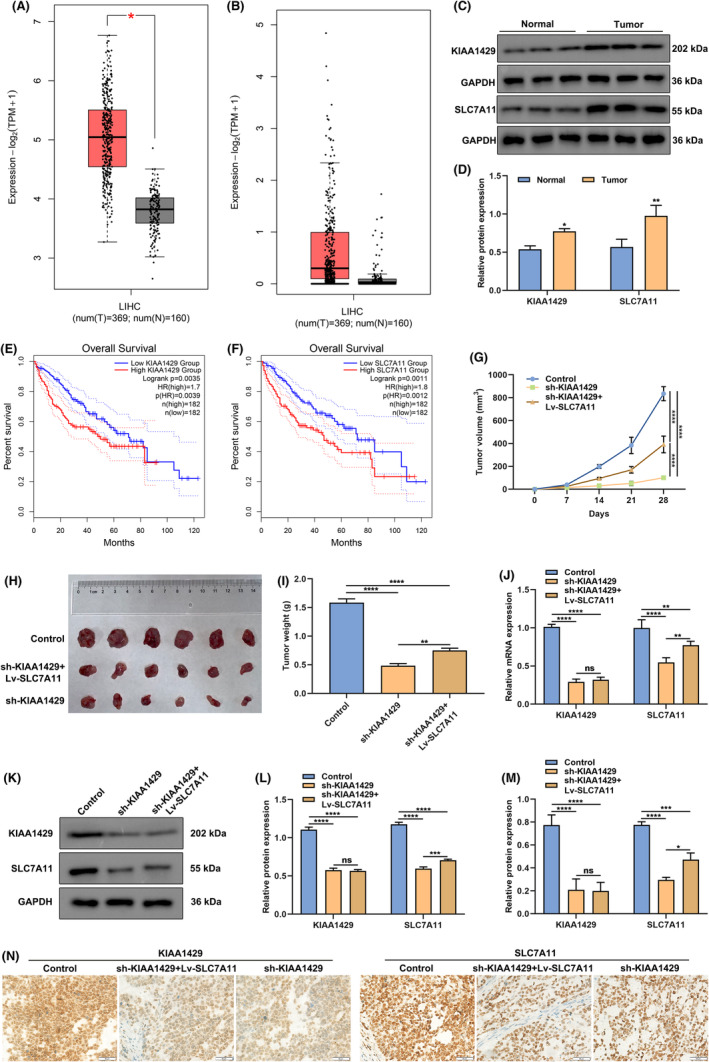
KIAA1429 inhibition mitigates HCC growth in vivo through SLC7A11. (A, B) KIAA1429 and SLC7A11 mRNA levels in HCC (*n* = 369) and normal specimens (*n* = 160) in TCGA‐LIHC cohort. (C, D) Protein levels of KIAA1429 and SLC7A11 in three paired HCC and normal specimens. (E, F) Overall survival of TCGA‐LIHC patients stratified by the median value of KIAA1429 and SLC7A11 mRNA levels. (G) Tumour growth curve of subcutaneous xenograft models following inoculation in the control, sh‐KIAA1429 and sh‐KIAA1429 + Lv‐SLC7A11 groups. (H) Representative subcutaneous xenograft tumours following 28‐day inoculation in the three groups. (I) Tumour volume following 28‐day inoculation in the three groups. (J) RT‐qPCR for detection of KIAA1429 and SLC7A11 mRNA levels in subcutaneous xenograft tumours from the three groups. (K–N) Western blot and immunohistochemistry for detecting protein levels of KIAA1429 and SLC7A11 in subcutaneous xenograft tumours from the above groups. Scale bar, 50 μm. *p < 0.05; **p < 0.01; ***p < 0.001; ****p < 0.0001; ns: no significance.

Up‐regulated KIAA1429 and SLC7A11 mRNAs were demonstrated in tumours in contrast to normal specimens across TCGA‐LIHC patients (Figure 7A,B). The similar expression patterns of KIAA1429 and SLC7A11 were observed at the protein level (Figure 7C,D). In addition, up‐regulated KIAA1429 and SLC7A11 were linked with worse overall survival outcomes (Figure 7E,F). Subcutaneous xenograft models were established for confirming the effect and mechanism of KIAA1429 on HCC growth. Consequently, sh‐KIAA1429 notably mitigated tumour growth and weight, which was partially reversed by Lv‐SLC7A11 (Figure 7G–I). Moreover, mRNA and protein levels of KIAA1429 and SLC7A11 were verified in subcutaneous xenograft tumours. In contrast to control group, KIAA1429 and SLC7A11 levels were observably lowered in sh‐KIAA1429 group (Figure 7J–N). Lv‐SLC7A11 did not affect sh‐KIAA1429‐mediated KIAA1429 inhibition but elevated SLC7A11 levels. In summary, KIAA1429 inhibition may attenuate HCC growth in vivo through SLC7A11.

## 4 DISCUSSION

Hepatocellular carcinoma remains the dominant histological form of primary liver cancer.^24^ Delayed detection, treatment resistance, high post‐treatment relapse risk, and molecular heterogeneity contribute to high mortality of HCC.^25^ Due to resistance of HCC chemotherapy together with high fatality rate, molecular mechanisms and genetic alterations of HCC progression are required for improving precision prevention strategies as well as survival prognosis of HCC patients.^26^ Our work illustrated a novel function of KIAA1429 in HCC and preliminarily unveiled its m^6^A‐dependent post‐transcriptional modification of SLC7A11 in ferroptotic cell death.

KIAA1429 is a key component of the m^6^A methyltransferase complex, and evidence has proven its tumorigenic function. For instance, KIAA1429 induces the resistance of non‐small‐cell lung cancer to gefitinib.^27^ It serves as an oncogenic mediator in breast cancer by modulating CDK1 with a m^6^A‐independent manner.^28^ Consistently, our study confirmed that suppressing KIAA1429 could induce tumour cell death. Ferroptosis escape mediated by oncogenes or oncogenic signalling triggers HCC initiation, progression, metastases, treatment resistance, etc.^29^ For example, Zhou et al.^29^ proposed that QSOX1 facilitates sorafenib‐induced ferroptosis in HCC by triggering EGFR endosomal trafficking together with inactivating NRF2. COMMD10 attenuates HIF1α/CP to strengthen ferroptosis and radiosensitivity by destroying Cu‐Fe balance in HCC.^30^ Some tumour cells depend on the ferroptotic defence system to survive under a metabolic and oxidative stress condition.^31^ Thus, destruction of the defence system may be lethal to such tumour cells, sparing normal cells. Altogether, ferroptosis might represent a potentially targetable vulnerability of cancer, and ferroptotic inducers have great potential in cancer therapy. The current study demonstrated that KIAA1429 inhibition induced ferroptotic cell death in HCC in accordance with increased cell death, iron and MDA levels, C11‐BODIPY‐positive cells, and ROS accumulation as well as reduced GSH level. The unique metabolism and high accumulation of ROS, together with specific mutations render some tumour cells inherently susceptible to ferroptosis, thus exposing a vulnerability to be therapeutically targetable. Ferroptosis inhibitors Fer‐1 and Lip‐1 blocked KIAA1429 suppression‐mediated ferroptotic cell death of HCC. Altogether, KIAA1429 contributed to HCC progression partially through modulating ferroptosis.

Several tumourigenic signalling pathways control the ferroptosis of tumour cells, and ferroptosis participates in the activities of several tumour suppressors as a natural barrier to tumour development. For instance, targetable LIFR‐NF‐κB‐LCN2 signalling may control liver tumorigenesis and is vulnerable to ferroptosis.^32^ O‐GlcNAcylation heightens sensitivity to RSL3‐mediated ferroptotic cell death through YAP/TFRC signalling in HCC.^33^ GSTZ1 may sensitise HCC cells to sorafenib‐triggered ferroptosis through mitigating NRF2/GPX4 signalling.^34^ In the present study, KIAA1429 overexpression observably heightened the activity of SLC7A11. In addition, SLC7A11 partially hindered KIAA1429 suppression‐mediated ferroptotic cell death of HCC cells. The modulation SLC7A11 by KIAA1429 was mitigated through m^6^A modification inhibitor cycloleucine. M^6^A‐RIP proved the binding of KIAA1429 to m^6^A on SLC7A11 mRNA transcript. More importantly, KIAA1429 suppression attenuated HCC growth in subcutaneous xenograft mice via modulating SLC7A11. Hence, KIAA1429 may protect HCC cells from ferroptosis with a m^6^A‐dependent post‐transcriptional mechanism of SLC7A11.

Previous research has proposed that KIAA1429 results in HCC progression with m^6^A medication mechanisms.^9^ Nonetheless, no literature reported the interaction of KIAA1429 with ferroptosis in HCC cells as well as the mechanisms by which KIAA1429 modulates ferroptosis. The current study revealed a novel regulatory mechanism of KIAA1429‐mediated m^6^A modification in ferroptotic cell death. In addition, we determined KIAA1429 as a novel ferroptotic inhibitor, and KIAA1429 suppression directly resulted in ferroptosis of HCC cells. KIAA1429 may protect HCC cells from ferroptosis through a positive regulation of SLC7A11 expression. Altogether, KIAA1429 inhibition in combination with ferroptotic induction therapy might become a potent therapeutic option of HCC in the future.

## 5 CONCLUSION

Collectively, the current study demonstrated that KIAA1429 inhibition induced ferroptotic cell death in HCC cells. KIAA1429 served as a ferroptosis inhibitor via modulating a key ferroptosis defence gene SLC7A11 with a m^6^A posttranscriptional modification mechanism. Hence, targeting KIAA1429 and KIAA1429‐mediated m^6^A modification of SLC7A11 might become a possible therapeutic regimen of HCC.

## AUTHOR CONTRIBUTIONS

**Hou hong Wang:** Data curation (equal); formal analysis (equal); writing – original draft (equal). **Wen li Chen:** Investigation (equal); methodology (equal); validation (equal). **Ya Yun Cui:** Software (equal); validation (equal); writing – original draft (supporting). **Hui hui Gong:** Data curation (equal); methodology (equal); supervision (equal). **Heng Li:** Conceptualization (equal); formal analysis (equal); project administration (equal); resources (equal); supervision (equal); writing – review and editing (equal).

## FUNDING INFORMATION

This work was funded by Project of Bozhou Municipal Health Commission (bzwj2022a001); Project of Bozhou Science and Technology Bureau (bzzc2022008); Project of the Affiliated Bozhou Hospital of Anhui Medical University (by2022001, by2023001).

## CONFLICT OF INTEREST STATEMENT

The authors declare no conflicts of interest.

## CONSENT FOR PUBLICATION

All patients provided written informed consent.

## Data Availability

The datasets analysed during the current study are available from the corresponding author on reasonable request.
